# The Holloway Sanatorium Inquiry

**Published:** 1895-03-16

**Authors:** 


### THE HOLLOWAY SANATORIUM INQUIRY.

The Home Office Inquiry into the case of the patient Weir, who died last September at Holloway Sanatorium, was concluded on the 12th inst., and the report will be prepared by Mr. Gully, Q.C., M.P., who has been assisted in his investigations by Dr. George Henry Savage. The patient was found in a dying state, confined by an apparatus known in the establishment as "the Dry Pack," which is thus described by Dr. Phillips: "The apparatus consists of a blanket and five broad straps connected at intervals by loops with two strips of webbing about six feet in length. The patient is laid upon the blanket, which is drawn over him and folded, so as to envelop him tightly from head to foot. He is then laid upon one of the straps of webbing and the other is brought down the centre of the front of his body, and the straps are drawn sufficiently tight to restrain the movement of all his limbs and keep his arms close to his sides. The upper part of the blanket is then turned back to prevent interference with respiration by the nose and mouth, and the two lower ends of the webbing are tied between the feet." The evidence of the doctor and attendants was taken respecting Weir's condition of "acute mania," and also with regard to the method and duration of the restraint employed, which had been adversely criticised by the Coroner's jury, and also by the Lunacy Commissioners, who had conducted an inquiry into the case.

